# Cases of Longevity No Test of the Average Duration of Man’s Life

**Published:** 1844-07

**Authors:** 


### SELECTIONS

FROM

### AMERICAN AND FOREIGN JOURNALS.

---

*Cases of Longevity no test of the average duration of man's life.*—Some constitutions are found to resist vaccine matter. Here and there, constitutions appear which resist all the noxious influences by which they are surrounded, and attain extreme old age. Not unfrequently we find the existence of these solitary individuals referred to as proofs of the general salubrity of the very circumstances under which generations have fallen and been buried around them. It is a singular fact, as yet unexplained, that the greatest proportion of centenarians are of the laboring classes; and that instances of them have from time to time appeared amidst the crowded populations in some of the worst neighborhoods of London, where the average duration of life is the lowest. It is remarked by M. Mallet, that in Geneva extreme old age has not participated in the prolongation of life which has taken place in the less advanced ages. In the periods of from 60 to 70 years of age, the amelioration is considerable; after 70 years, there is no perceptible improvement; after 80 years, the aged have, indeed, a little less probability of life at the present time than they had in the 16th century. Centenarians, who were not rare in the 16th and 17th centuries, now disappear; during the last 27 years Geneva has not produced a single one.—*Sanitary Condition of the Laboring Population of Great Britain.*

